# Nanobody Paratope Ensembles in Solution Characterized by MD Simulations and NMR

**DOI:** 10.3390/ijms23105419

**Published:** 2022-05-12

**Authors:** Monica L. Fernández-Quintero, Eugene F. DeRose, Scott A. Gabel, Geoffrey A. Mueller, Klaus R. Liedl

**Affiliations:** 1 Department of General, Inorganic and Theoretical Chemistry, Center for Molecular Biosciences Innsbruck (CMBI), University of Innsbruck, Innrain 80-82, A-6020 Innsbruck, Austria; monica.fernandez-quintero@uibk.ac.at; 2 Genome Integrity and Structural Biology Laboratory, National Institute of Environmental Health Sciences, 111 T.W. Alexander Dr. MD-MR-01, Research Triangle Park, NC 27709, USA; derose@niehs.nih.gov (E.F.D.); gabel@niehs.nih.gov (S.A.G.)

**Keywords:** single-domain antibody, nanobody, NMR, molecular dynamics simulations

## Abstract

Variable domains of camelid antibodies (so-called nanobodies or V_H_H) are the smallest antibody fragments that retain complete functionality and therapeutic potential. Understanding of the nanobody-binding interface has become a pre-requisite for rational antibody design and engineering. The nanobody-binding interface consists of up to three hypervariable loops, known as the CDR loops. Here, we structurally and dynamically characterize the conformational diversity of an anti-GFP-binding nanobody by using molecular dynamics simulations in combination with experimentally derived data from nuclear magnetic resonance (NMR) spectroscopy. The NMR data contain both structural and dynamic information resolved at various timescales, which allows an assessment of the quality of protein MD simulations. Thus, in this study, we compared the ensembles for the anti-GFP-binding nanobody obtained from MD simulations with results from NMR. We find excellent agreement of the NOE-derived distance maps obtained from NMR and MD simulations and observe similar conformational spaces for the simulations with and without NOE time-averaged restraints. We also compare the measured and calculated order parameters and find generally good agreement for the motions observed in the ps–ns timescale, in particular for the CDR3 loop. Understanding of the CDR3 loop dynamics is especially critical for nanobodies, as this loop is typically critical for antigen recognition.

## 1. Introduction

Camelids such as camels, dromedaries, llamas, alpacas, guanacos, and vicuñas contain heavy-chain-only antibodies, which consist of a stable and soluble single-antigen-binding variable domain [1,2,3]. Single-domain antibodies (V_H_Hs), also known as nanobodies^®^, have received increasing attention as highly versatile proteins with a high affinity for a variety of targets, and their flexibility has opened the door for a new generation of therapeutics [4,5]. Nanobodies have been proposed as treatments for various diseases and infections, including: autoimmune diseases, allergies, and for use as antivirals. The term nanobody originates from a trademark introduced by the company Ablynx in 2003 and became a general classification for these single-immunoglobulin domain proteins, reflecting their small size compared to antibodies, which are more than 10 times larger [6]. Nanobodies are potent alternatives to conventional antibodies, because of their small size, refolding capacity, stability, specificity, and natural origin [4,7,8]. Structurally, nanobodies are still functional without a light-chain counterpart found in typical antibodies, as they lack the hydrophobic interface, which is usually required to pair with a light chain in IgG-type antibodies [9]. Thus, the amino acid sequences of naturally occurring V_H_H antibodies are expected to contain adaptations to compensate for the absence of the paired light-chain variable domain. Nevertheless, it has been shown that V_H_H sequences share a high degree of similarity (~80%) with conventional human variable heavy-chain domains (V_H_). However, the less hydrophobic interface in V_H_H originates from several residue substitutions (mainly hydrophilic residues), namely L11S, V37F/Y, G44E, L45R/C, and W47G (following the Kabat nomenclature [10]), that discriminate the conventional V_H_ from V_H_H. These residue substitutions are believed to enhance the stability in the absence of the light chain and result in favorable biophysical characteristics, such as stability and low aggregation risk [11,12]. However, the amino acid residues at the positions that determine the typical immunoglobulin fold are all well-conserved in the V_H_H [13,14].

**Figure 1:**
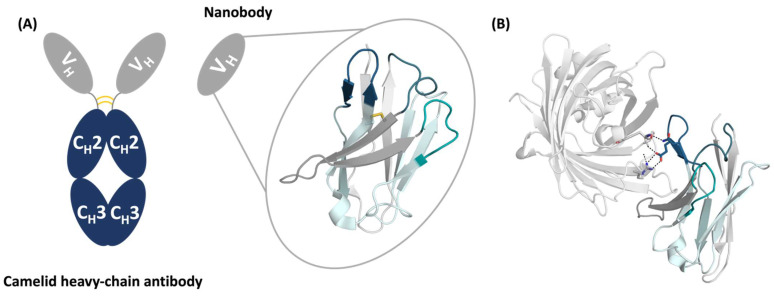
Structure of the anti-GPF nanobody with and without the antigen present. (**A**) Schematic representation of a heavy-chain-only antibody and a nanobody with the structure (PDB accession code: 3OGO). The CDR1, CDR2, and CDR3 loops are colored in teal, deep-teal, and dark blue, respectively. The conserved disulfide bridge is indicated in yellow. The FR1, FR2, FR3, and FR4 are illustrated in light grey, dark-grey, aquamarine, and turquoise, respectively. (**B**) Structure of the anti-GFP binding nanobody in complex with GFP. The dashed lines show interactions of the CDR3 loop with the antigen.

The V_H_H domain consists of four framework regions (FR1, FR2, FR3, FR4), which are separated by three hypervariable loops, known as the complementarity-determining region loops (CDRs), namely the CDR1, CDR2, and CDR3 loops (Figure 1). The antigen-binding site, the paratope, is formed not exclusively by the CDR loops but also by neighboring framework residues, which contribute to recognizing and binding the antigen. V_H_Hs contain a canonical disulfide bond connecting the β-strands of framework regions 1 and 3. Various camelid antibodies also have an additional disulfide bond connecting either the end of the CDR1 loop with the CDR3 loop (camels) or the beginning of the CDR2 loop with the CDR3 loop (llamas). Furthermore, the CDR3 loop of V_H_Hs can be substantially longer compared to conventional IgG and possesses the unique ability to form long extensions to reach cavities and buried binding sites with high shape complementarity [15]. Thus, the CDR3 loop of V_H_Hs plays a critical role in recognizing and binding the antigen [16]. However, due to the high diversity in the length, sequence, and structure of the CDR3 loop, structure prediction remains challenging [17]. Recent studies using molecular dynamics (MD) simulations found that one single static structure is not sufficient to functionally understand the antigen-binding site, and suggested to characterize the paratopes as ensembles in solution [18,19,20]. We previously showed that antigen recognition follows a conformational selection-type binding to the dominant structure in solution, which is frequently not reflected by a single structure due to crystal packing effects in the apo form [18,21].

MD simulations are a powerful tool to study biomolecular movements in atomistic detail on timescales ranging from picoseconds to milliseconds. Various studies have already validated MD simulations against quantitative experimental data of protein structure and dynamics to further improve the MD method itself, as well as to mechanistically interpret the experimental data [22,23,24]. NMR data of proteins in solution are uniquely suited to observe different timescales of protein dynamics and to assess the quality of protein MD simulations [25,26,27]. In this study, we tested the applicability of recently honed MD methods to characterize the conformational diversity of an anti-GFP binding nanobody by combining molecular dynamics simulations with experimental data from NMR spectroscopy in order to better understand nanobody structures and dynamics.

## 2. Results

**Figure 2:**
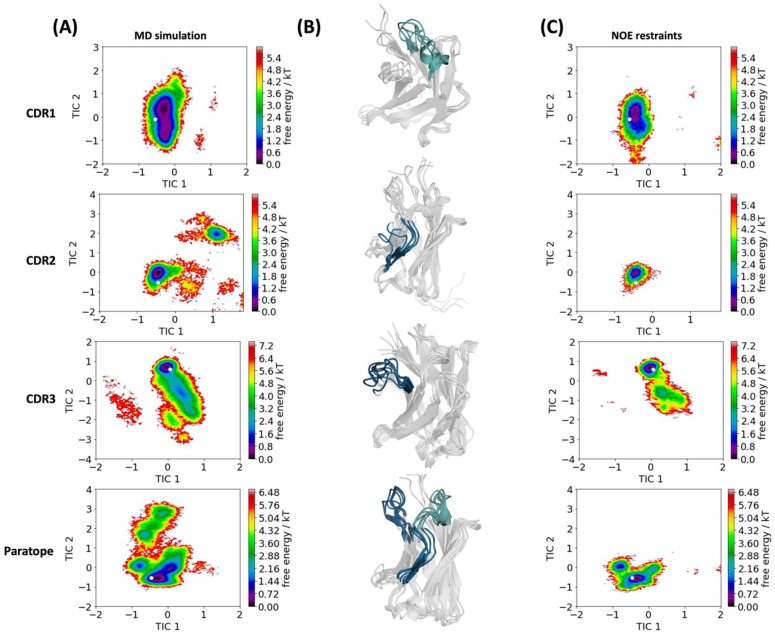
Free energy surfaces of the simulated MD ensemble (**A**) and the simulated NMR ensemble (**C**). Combined tICA of the simulated MD ensemble and the simulated NMR ensemble was performed to generate kinetic coordinate systems for each CDR loop and the whole paratope. tIC1 and tIC2 represent the directions of the two slowest movements of the system. The crystal structure is projected into these coordinate systems and illustrated as a white dot. (**B**)The respective structure representatives of the simulated MD ensembles are shown in the middle, focusing on the respective CDR loops.

To characterize the conformational diversity of the CDR loops of the anti-GFP nanobody [28,29] in solution, we used a protocol using bias-exchange meta-dynamics in combination with classical MD simulations (resulting in 15.4 µs of MD simulations) to overcome the limitations of conformational sampling imposed by high-energy barriers [19,30,31]. These simulations will be referred to as “simulated MD ensemble”. Additionally, we used experimental nuclear magnetic resonance (NMR) nuclear Overhauser effect (NOE) data in combination with molecular dynamics simulations to understand the dynamics and the respective timescales of conformational rearrangements for the anti-GFP nanobody [28,29]. The time averaging and 1/r^6^ dependence of the interatomic distances on intensity provides accurate information for assessing MD sampling. The simulation performed with NOE restraints will be referred to as “simulated NMR ensemble”. Figure 1 illustrates the structure of the nanobody, highlighting the three CDR loops, and displays the complex structure of the nanobody with GFP. Figure 1B shows that the CDR3 loop plays a critical role in antigen recognition and binding, while the CDR1 and CDR2 loops do not interact with the antigen. To visualize the conformational spaces and to reconstruct the free energy landscape of the CDR1, CDR2, and CDR3 loops, and the paratope, we performed a time-lagged independent component analysis [32] (tICA) of the accumulated 15.4 µs of MD simulations and the simulation including the time-averaged NOE restraints, based on the backbone torsions of the respective CDR loops in the same coordinate system. The free energy surfaces and the respective conformational ensembles are illustrated in Figure 2. What can immediately be seen is that the conformational space is confined to a single dominant minimum, revealing structural rearrangements in the low-nanosecond timescale. The transition timescales have been estimated based on the tICA free energy landscape, which takes the number of conformational transitions into account [33]. In agreement with our observations from the simulated MD ensemble (Figure 2), we found by comparing our results with the NMR ensemble (including the time-averaged NOE restraints) that most of the movements occurred in the ps–ns timescale. Additionally, we also performed MD simulations with NOE restraints to compare the respective ensembles in solution (Figure 2). While we covered a very similar conformational space, the observed dynamics including the NOE restraints resulted in a more restricted conformational space.

The NOE restraints were obtained with the following strategy. Since a crystal structure was already known, the goal was not to test the limits of NMR structure techniques, but rather to provide as many calibrated NOEs as accurately as possible. To do this, 171 NOEs were manually assigned with the aid of the crystal structure 3OGO [28]. A round zero of structure calculations provided a loose ensemble of starting structures that were used by CYANA to assign the remaining NOEs using an iterative strategy. A total of 1194 upper-limit restraints were determined by CYANA.

**Figure 3:**
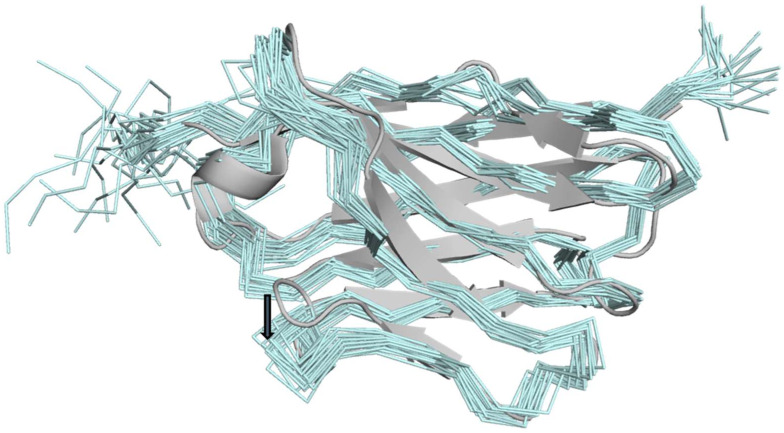
NMR ensemble compared to 3OGO. The NMR ensemble of 20 structures calculated by CYANA (aquamarine) is compared to the crystal structure 3OGO (grey). A dark-grey arrow indicates the displacement of residues 57–69.

The CYANA calculated structures had 1.5 Å RMSD for the residues 3–113. Table 1 shows that Ramachandran space was never violated, only a handful of NOEs were occasionally violated in the ensemble, and eight van der Waals close contacts were noted. These statistics show that the NMR restraints are not in conflict with standard protein geometry. Figure 3 shows that the NMR ensemble matches the 3OGO structure well (Figure 3), with 1.4 Å RMSD over 110 Cα-atoms, indicating that the accuracy of the ensemble is on a par with the precision. The biggest difference in the NMR versus the 3OGO crystal structure is in the displacement of the exterior loop residues 57–69. In this loop, the backbone angles are very similar, but the NMR loop is slightly displaced from the beta sheets of the nanobody, likely due to a paucity of long-range restraints on the exterior of the protein. The structure demonstrates that the upper-limit restraints calibrated from the NOE intensities accurately reflect the nanobody structure, as determined by crystallography.

**Figure 4:**
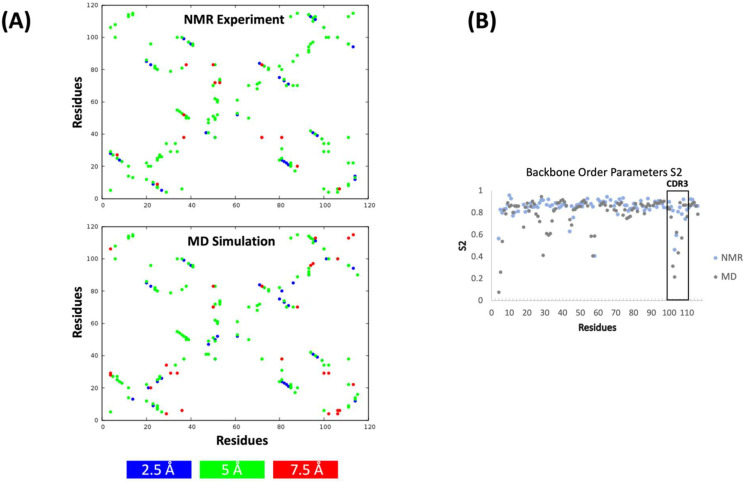
Calculated and experimentally measured NOE distances and order parameters (S^2^) for the anti-GFP nanobody. (**A**) Experimental upper NOE limits are shown on top, calculated NOE limits are shown at the bottom. (**B**) Experimental S^2^ and calculated S^2^ are colored in light-blue and grey, respectively.

Apart from directly comparing the obtained conformational spaces (Figure 2), we also compared the experimental NOE upper limits with the calculated NOEs from the simulated MD ensemble (Figure 4A). The results show the presence of highly similar NOEs between the simulated MD ensemble and the experiment. To compare the order parameters (S^2^) from our simulated MD ensemble with the experimentally determined ones, we performed 1 µs of classical MD simulations by using the dominant paratope ensemble structure as a starting structure. We calculated S^2^ from the obtained classical MD trajectory by using pytraj [34] and present the comparison with the experimentally determined order parameters in Figure 4B. While we found few residues in the CDR1 loop with dynamic discrepancies, we observed an overall good agreement between the calculated and experimentally measured order parameters.

## 3. Discussion

In this study, we characterized the conformational diversity of an anti-GFP nanobody [28] with molecular dynamics simulations and NMR. Figure 2 compares the conformational landscapes of the simulated MD ensemble and simulated NMR ensemble for the individual CDR loops and the whole paratope in a combined coordinate system. We observe that the NOE restricted MD simulation results in a decidedly more restricted conformational space compared to the simulated MD ensemble trajectories. It is therefore worth discussing any bias in the method to measure and calibrate the NOE restraints. The iterative assignment and calibration of NOEs by CYANA is designed to converge on a single structural ensemble with a low RMSD, as incorrect assignments and erroneous distances are removed. This strategy mimics manual NMR analysis and other auto-assignment methods. The point is that rare conformations that might be present in the NOE data are likely pruned, and the resulting structures are closer to a global minimum. Therefore, it is reasonable that the ensembles restrained by NOEs are a narrow subset of the simulated MD ensemble. Attempts to include rare conformations in NMR structure calculations utilize the measurement of exact NOEs (eNOEs) and better algorithms that account for spin diffusion and multiple states [35,36]. However, measuring exact NOEs requires significantly more spectrometer time (up to six additional 3D spectra are recommended [35]) and the methods are not as amenable to automated assignment. Enhanced sampling with classical MD simulations in addition to traditional NMR structure analysis likely represent a viable alternative procedure to examine rare states.

In comparison to various antibodies [20,37] and other single-domain formats (e.g., VNARs, T-cell, and β-variable domains) [31,38,39,40], this anti-GFP nanobody shows limited conformational diversity, indicating a high specificity for the GFP protein. Even though this antibody already seems quite matured, describing the antigen-binding site as a conformational ensemble in solution is necessary to provide a quantitative understanding of the binding interface [38].

Rare states can also be detected by NMR on timescales of ms to sub-ms [41]. However, the CPMG experiments did not detect any convincing dispersions (data not shown). On a faster timescale of ns–ps, NMR can directly assess molecular motions on fast timescales. Comparison of these measurements with MD is common and generally shows good correlations between measured and simulated order parameters [42,43]. Similarly, in the case of this nanobody, there is generally good agreement between the measured and calculated order parameters, especially for the CDR3 loop, again suggesting that our MD simulations capture realistic molecular motions. Capturing the flexibility of the CDR3 loop is particularly important for nanobodies, as this loop is mainly involved in recognizing and binding to the antigen [12,15,16,44]. Admittedly, there were very few residues in the CDR1 loop with significant dynamic excursions, so the analysis of large motions is slightly biased toward a few interesting loop residues. Figure 4A shows the calculated and experimentally determined NOEs and confirms the good agreement of both methods. In contrast to other single-domain antibodies, which showed substantial conformational rearrangements in the micro-to-millisecond timescale, this anti-GFP nanobody is confined to one single minimum with structural changes in the ps–ns timescale [31,38].

## 4. Methods

### 4.1. Structure Preparation

As starting structures for our simulations, we used the crystal structure of the anti-GFP nanobody with the PDB accession code 3OGO [28].

The starting structure was prepared and protonated in MOE using the Protonate3D tool [45,46]. Charge neutrality was ensured by using the uniform background plasma approach in AMBER [47,48]. Using the tleap tool of the AmberTools20 package, the crystal structure was soaked in cubic water boxes of TIP3P water molecules with a minimum wall distance of 10 Å to the protein [47,49,50,51]. The structures were described with the AMBER force field 14SB [52]. The nanobody was carefully equilibrated using a multistep equilibration protocol [53,54].

### 4.2. Metadynamics Simulations

To enhance the sampling of the conformational space, we performed 500 ns of well-tempered bias-exchange meta-dynamics simulations in GROMACS with the PLUMED 2 implementation [55,56,57]. As an enhanced sampling technique, we chose meta-dynamics because it allowed us to focus the enhanced sampling on predefined collective variables (CV) [58,59,60]. The sampling is accelerated by a history-dependent bias potential, which is constructed in the space of the CVs. As collective variables, we used a well-established protocol, boosting a linear combination of sine and cosine of the ψ torsion angles of all six CDR loops calculated with functions MATHEVAL and COMBINE implemented in PLUMED 2 [21,39,61]. As discussed previously, the ψ torsion angle comprehensively captures conformational transitions [62]. The underlying method presented here has been validated in various studies against a large number of experimental results. The simulations were performed at 300 K in an NpT ensemble to be as close to the experimental conditions as possible and to obtain the correct density distributions of both protein and water. We used a Gaussian height of 10.0 kJ/mol and a width of 0.3 rad. Gaussian deposition occurred every 1000 steps and a bias factor of 10 was used. The resulting trajectory was clustered with the program cpptraj using the average linkage hierarchical clustering algorithm with a root mean square deviation cutoff criterion of 1.2 Å, resulting in a large number of clusters [63]. The cluster representatives for the nanobody were equilibrated and simulated for 100 ns using the AMBER 20 simulation package [47].

### 4.3. Molecular Dynamics Simulations

MD simulations were performed in an NpT ensemble using the pmemd.cuda module of AMBER 20 [64]. Bonds involving hydrogen atoms were restrained with the SHAKE algorithm, allowing a timestep of 2.0 femtoseconds. Atmospheric pressure (1 bar) of the system was set by weak coupling to an external bath using the Berendsen algorithm [65]. The Langevin thermostat was used to maintain the temperature during simulations at 300 K [66,67].

With the obtained trajectories, we performed a time-lagged independent component analysis (tICA) using the python library PyEMMA 2 [68], employing a lag time of 10 nanoseconds. tICA was applied to identify the slowest movements of nanobody CDR loops and consequently to obtain a kinetic discretization of the sampled conformational space.

### 4.4. NOE Restraints Simulations—NMR Ensemble

The NOE distances were derived from intramolecular magnetic interactions which are inversely proportional (1/r^6^) to the distances between protons. The NOE values were utilized as determined by CYANA, see below. The structure was minimized, equilibrated, and then simulated for 1 μs using the available 1194 NOE distance restraints, including time-averaged constraints in an NpT ensemble using pmemd.cuda, following the same parameters as described in the “Molecular Dynamics Simulations” Section [69,70]. A time constant for the memory function for the distance restraints of 100 ps was chosen.

### 4.5. Expression

BL21(DE3) *E. coli* were grown to an OD ~4 in 1 L of 2XYT media at 37 °C. Cells were pelleted, then brought up in 1 L of M9 minimal media which was labeled with either ^15^NH_4_Cl or ^15^NH_4_Cl and U-^13^C-glucose. Cells had a 1 h equilibration time while shaking at 25 °C. The temperature was lowered to 18 °C and cells were induced with 0.5 mM of IPTG. Protein was expressed overnight (12–18 h). Cells were pelleted and frozen for later purification. The expressed protein sequence was: MAQVQLVESGGALVQPGGSLRLSCAASGFPVNRYSMRWYRQAPGKEREWVAGMSSAGDRSSYEDSVKGRFTISRDDARNTVYLQMNSLKPEDTAVYYCNVNVGFEYWGQGTQVTVSSHHHHHH.

### 4.6. Purification

Frozen cells were thawed and re-suspended in 15 mL of a lysis buffer containing 1 M NaCl, 50 mM HEPES (pH 7.6), 10 mM imidazole, 1 mM MgSO4, 18 mM B-mercaptoethanol, 1 mL B-PER (Thermo Scientific), and an EDTA-free protease inhibitor cocktail tablet (Roche). Cells were lysed by sonication (3 cycles × 2 min/cycle) on ice. The lysate was centrifuged at 28,000× *g* for 30 min at 4 °C. The supernatant was syringe-filtered to produce a clear lysate. The lysate was loaded onto an IMAC column packed with Ni^+2^-charged NTA-resin (Amersham). Protein was then eluted from the column with a buffer consisting of 0.5 M NaCl, 50 mM HEPES (pH 7.6), and a stepped gradient of imidazole of 10, 25, and 300 mM, where the protein eluted in the 300 mM imidazole fraction. The protein was further purified using a Superdex 26/60 S75 preparative-grade gel filtration column (GE Amersham) with a buffer consisting of 150 mM NaCl, 25 mM HEPES (pH 7.4), 1 mM DTT, and 0.25 mM sodium azide.

### 4.7. NMR Data Acquisition

The NMR backbone and side-chain peaks were assigned using standard NMR techniques [71]. The peaks were remarkably well-dispersed, facilitating a straightforward attribution of spectral coordinates to individual atoms. The NOE spectra were analyzed in conjunction with the 3OGO structure, which allowed the rapid assignment of the beta-strand interactions that were used to assign H-bond pseudo-NOEs and other long-range NOEs. A total of 38 H-bonds and 171 NOEs were input as initial restraints for structure calculations with CYANA [72]. The H-bond H–O interactions were set with upper limits of 2.5 Å, and the NOE upper limits were set at 5 or 7 Å for leucine or valine methyl. These ‘round-zero’ structures had, as expected, low convergence, with a 3–4 Å RMSD. The 10 lowest energy round-zero structures were used as initial structures in a subsequent round of CYANA calculations that included all the unassigned peaks (4224) and the manually assigned NOEs. The peak intensities and ppm coordinates were converted to NOE upper limits in CYANA’s iterative structure determination approach. The NOE upper limits (1194) after the final iteration of CYANA were used for comparison with the MD trajectories. Details of the structural convergence and structure quality are shown in Table 1.

Relaxation data were acquired using common pulse sequences for T1, T2, {^1^H-^15^N}-NOE, and relaxation dispersion at 600 and 800 MHz [41,73,74]. The T1, T2, and {^1^H,^15^N}-NOE data were analyzed using the program RELAX to choose the best motional model for order parameters [75,76]. Relaxation dispersion data were analyzed with GUARDD [77].

## 5. Conclusions

We showed that the CDR loop ensembles obtained with and without time-averaged NOE restraints were in good agreement, which was reflected in the very similar covered conformational space, with the same dominant minimum in solution. In line with the NMR data, we observed that most of the movements for the CDR loops of this nanobody occurred in the low-nanosecond timescale. This is also in line with the good accordance of the order parameters, especially for the CDR3 loop. Thus, we showed that our MD ensemble characterizes and captures the relevant CDR loop conformations in solution that are important for antigen recognition.

## Figures and Tables

**Table 1:** Summary of restraints used for NMR structure determination and structure refinement statistics (20 structures) of experimental restraints.

NOE Assigned by CYANA	1194
Manually assigned NOEs	171
H-Bond restraints	38
Dihedral restraints	194
Backbone RMSD (residues 3–113)	1.5 Å
**Ramachandran Space**	
Most Favored	80.3 %
Additionally Allowed	19.2 %
Generously Allowed	0.5 %
Disallowed	0
**Violations**	
NOE (<0.2 Å)	19
Dihedrals	3
VDW close contacts	8

## Data Availability

The PDB code for this deposition is 7V0V. The BMRB code for this deposition is 31020.
